# Supplementary material for: Patterns of Intron Gain and Loss in Fungi
Source: PLoS Biol. 2004 Nov 30;2(12):e422. doi: 10.1371/journal.pbio.0020422 (PMC532390; doi:10.1371/journal.pbio.0020422)
Supplement: Table S1 — Also available at http://genes.mit.edu/NielsenEtAl/. (4.3 MB ZIP). [file pbio.0020422.st001.zip › NielsenEtAl/html/576.html]

AN6733.1.NCU05242.1.MG01776.1.FG09770.1


```
 CLUSTAL W (1.82) Multiple Sequence Alignments - Introns Inserted


Sequence 1: NCU05242.1	1033 aa
Sequence 2: FG09770.1	992 aa
Sequence 3: MG01776.1	1042 aa
Sequence 4: AN6733.1	1003 aa
Alignment Length: 1090 aa
Number Identitical Residues: 316 aa
Alignment Score (without introns) 17901


MG01776.1 	MASAAVPSCATATLILDDAEQLKPRS---LSNGNMFQGQEPTPPHTIKQEDS-DRARPRS
NCU05242.1	----------MTSVLPSQSSDIKPPHSDCLPDGAMPQYQEPTPPHNIKQEDEQERGRRRS
FG09770.1 	----------------MGTQNISPRD----------------------TSDVSARGRRRS
AN6733.1  	--------------MAFAVTPVAPLS----------------------------------
          	                     : *                                    

MG01776.1 	QSHDLDEDDKRDLKQVTPDDGAQDPENSSHSDGGPARKRRRSRK-GLDKKFECSEEGCGK
NCU05242.1	RSDDKDVEGL-DLEDMEDGDGNGNDAHSSNEEG-PARKRRRSRK-GLDKKFECPEQGCGK
FG09770.1 	RSPENDGHEQ-DPRGEGNQDGPNGASSNSDGPG-PARKRRRSRK-GLDKRFECSAEGCGK
AN6733.1  	-------------QGTRPDTEVGSESPDVPEANGPPRKRKRTRKVQVDRKFDCSYEGCGK
          	             .         .   .    ..*.***:*:**  :*::*:*. :****

MG01776.1 	SYSRAEHL2YRHQLNH~APKNIFYCGIGDCTRSFVRQDLCNRHKDRHTAKGSSLNRKDRD
NCU05242.1	SYSRAEHL2YRHQLNH~NPKQVYKCGIGDCQRTFVRLDLCNRHKERHTAKGSALSR--KD
FG09770.1 	SYSRAEHL2YRHQLNH~NSKQTYHCTFPNCTRTFVRGDLLKRHMDRHAAKGSQLNQ--RD
AN6733.1  	SYSRAEHL~YRHQLNH1APKQIYRCDFPECYRSFVRQDLCVRHRERHTTQGSQLQK--RD
          	******** *******  .*: : * : :* *:*** **  ** :**:::** *.:  :*

MG01776.1 	GFMPQPSSPFATGVSSAFPVPSPTSPADRRRSIPYPNAVP----QHVHYGSANPSFGQQT
NCU05242.1	SLMSLASPTTD-GRPPFMTQGSASP---ETNRLGNGKGRT----LSMQFNSPKDAMGSPY
FG09770.1 	SMAGHMGPVIAPGQPPNFSR---------NNSIDYGKSQP----TNMSYQTSQIPTPSPY
AN6733.1  	HFAQAASTSTGGIAKSQIVHTAVQLPQNAPPVLSPPDSKRGSTGPDQPIAASSIPVSSPT
          	 :    ..       . :   :   . .    :   ..  .::.      :.. .  .  

MG01776.1 	AVSPSTYSPTMRQNP-----LGYTQSPVTSTQAQPHSLPLHSSPGVHPRRPNVPP----D
NCU05242.1	TPMTASAPTNGYSNG------IDYMQHDAHYQHMAGQRSLQHSP-TGPQRPSVQT----N
FG09770.1 	SPMGN-APTGMYQNGPMPNGMETYMSPTQGYDNRTPQLSQPQSP-AMSQRPSISA----G
AN6733.1  	SRGFNRFSYQPAAQQHAPTAEVPYSQPCDLPNTPITTTTFNSPPLQRPLQVGSNTRHALN
          	:      .     :   ...     .     :      .   .*   . : .  .  : .

MG01776.1 	GSPYGVLSPVSTHNGFY-SHQNTPQ-TQI-FTAQNNFPPLDLSPISMSQATSPVIPPDLG
NCU05242.1	VGPYGVLSPVSTQPGYHGHSVNTSQ-SAVPYVTPQNFPPFSLPPSDFATTSPAAVVSREA
FG09770.1 	NSQYNVMSPIANQHGFP-NQQNVAQ-NSS-FISQQNVMPMNLPPAQYSSGPDSTVVT---
AN6733.1  	GSPTNELGPARSAALDEPLVSNRSQDLGASYAVSADLTGSLVSPSAYTDQAGLQIPVDGY
          	 .  . :.*  .         * .*.   .:    :.    :.*   :  .   :     

MG01776.1 	QSTPFATTAFQAEYSDQVGQQRGSDLMMLDQMTM---------PQTMPVFGSDNIYHKSP
NCU05242.1	QQQAYVP-LTTAESFDHHPQDTG-EIVRLDQMSM---------QQTMPVFGTDSILNKSP
FG09770.1 	----------SAEYSEPGTTQAS-EMMMLDQMSM---------PATVPVFGEGGDLNKSP
AN6733.1  	SDINMAP--VTSSASAPLDQTNGLTLDSMAGMAVGDMQFDGLNSCVYPVFGGES--NRSP
          	..    .    :.         .  :  :  *::.. . .. .  . ****  .  ::**

MG01776.1 	YAGMPEDFMAYLFNTGGSQPSDSPQGRIAP~TPRFSH2IPY-------EPSQLGFFP---
NCU05242.1	YVGMPEDFMAYLFNTHGEGSPTVVPVQGLN2YGEFST~NQY---PFYNDPSQMGYFPQMA
FG09770.1 	YVGMPEDFMAYLFNSPSQAMTPVMGPNYTN2YAELQG~NQFNVSLLGTDSNSLGYFP-TA
AN6733.1  	FH-MGDDFTAWLFNEPVPGSSMAPPANMVP~G--FMD~AQMQNQFLMSDPSYGNFLNSVI
          	:  * :** *:***      .     .       :      . .   .:..  .:: .  

MG01776.1 	-QQVMSVTNLLDQNLPESVISEEKSSEIFEYIKERFHGIESAPVERQKENIVEGDRSQPS
NCU05242.1	PQQIMSVENLLDQNLPESVISDPKSAEIFELIKERFHENGHTPIERQRDNILEGDRNNEN
FG09770.1 	PQQVMAVNNLLDQQVPEATISEEKSQELFDFIKERFHDNKHAPPDRQWADLIEGDRANGG
AN6733.1  	PAHPMSVTSILDPGSPRAIMSEEKRQELLDLMSTRFNEAAYSAVAKRKDALMDGDMDEDR
          	. : *:* .:**   *.: :*: *  *::: :. **:    :.  ::   :::**  :  

MG01776.1 	HMLSRQMMQAYIMSYWLHVSDQVPILHKPTFQPDKAQNLLLIAVMMIGAACLDKEHGPKI
NCU05242.1	HMLSRKMMQAYIVSYWLHFSDQIPILHKPTFSPDKTPNLLLIAMMTIGAACLDKAYGQKV
FG09770.1 	HMLSKRMMQAYIGSYWYHFSDQMPILHKPTFSPDRTPNLLLLAMMAIGGACLDRTHGHKV
AN6733.1  	HVLSLSKMQTYIGSYWYHFHAQLPILHRPTFVADKAPNLLLLAVIAIGAATLDSIHGQEV
          	*:**   **:** *** *.  *:****:*** .*:: ****:*:: **.* **  :* ::

MG01776.1 	RTAGAQLSNFLAWDLRWALFRDSQNFGPPAKLWVFQSLLLLEMYEKMYSTRQLHERAHIH
NCU05242.1	TKAGAELSNFLAWHLRWEMFQDP-NCRPPAKLWVFQTLLLLELYEKMYSTRELHERAHIH
FG09770.1 	TRASAQLSNFLAWHLRWDIFMDV-NFRPPAKLWIFQSLILLELYEKMFATRELHERAHIH
AN6733.1  	TEAASELADFIMWHLRWEIFMEG-DFRPPAKLWVFQALLLLEVYEKMYSTRALHERAHIH
          	  *.::*::*: *.*** :* :  :  ******:**:*:***:****::** ********

MG01776.1 	HATTITLMRRGRSLLGKSALDSPPTAQHG----SRQSSTSGVAHTPDEWWNHWITNESTR
NCU05242.1	HATTITLMRRGRALIGKSALDSPPNPRDDKTNGSRHSSTSGMAHTPDEWWNHWITNEATR
FG09770.1 	HATTITLMRRGRSLIGKSALDSPPNPRDG-SNGSRQSSTSSTTGTADKWWEHWVTNEATR
AN6733.1  	HDTTLTLMRRGSSLIGRHSFDSPASLRDDRQARSTTGSTMTPDFAADDSWAHWIKTEATR
          	* **:****** :*:*: ::***.. :..    *  .**     :.*. * **:..*:**

MG01776.1 	RAAFAAFLIDSIHGTIFGHNVVMVAHEMRLPLPCDDNLWKATSGAEVGRLEANLQSNNIK
NCU05242.1	RAAFAAFVIDSIHATMFGHSTVMVAHEMRLPLPCDDKLWKAKSSAEVGKIEAELMSQG-K
FG09770.1 	RAAFAAFIVDSIHATMFGHSAVMVAHEMRLPLPCDESMWRAGSSSEVWRIESNLLSNGIK
AN6733.1  	RVAFAAFVLDSTHATMFGHSAKMVAHELRLPLPCDEALWSATSASEVARVQASLHANGVR
          	*.*****::** *.*:***.. *****:*******: :* * *.:** ::::.* ::. :

MG01776.1 	PISFLEGLRRTLGNQPVQTNAFGRTVLIAGLLNVSYHMNQRDLQVYSLGRPALQSLGGRE
NCU05242.1	PIGFLDGLKRTLNGQTVQTNSFGRTILMAGLLSVSWHMNQRDLQVNSLG--ATTALGGRD
FG09770.1 	PTSFLEGLKRTLSGQDVHTTSFGRTVLMTGLLSVSWHMHQRDLQVNVLGGGIIQALGGRD
AN6733.1  	PVMFLDGLKRTLNGQRVRTNAFGRTILMAGLLSVSWHMNQRDLQVSSLG--VAHALGGRD
          	*  **:**:***..* *:*.:****:*::***.**:**:******  **     :****:

MG01776.1 	KWRQALRQAFDVWRDEFDKAIK--RGDIPSEVYLYGQDKHSEANVIFEGRTVLHSLAHMA
NCU05242.1	KWRTTLTRAFDSWKKEFDRALEEKRGDMA-DPYFFG-VRGNETDVVFESRVVLHHLAHMA
FG09770.1 	RWRATLTRAYDTWKADFDKHLE--RNEAA-DPYQDDSTKRQEFNVVFESRTVLHHLAHMA
AN6733.1  	KWRSALLRAFDNWRRDFDEALQP---GMASYPNGYRGRYALDEDNVFESRDVLHGLAHMA
          	:** :* :*:* *: :**. ::      .:           : : :**.* *** *****

MG01776.1 	MHVDIVDCQIYARAKRLLGRPIGTQDLNSSKRRMKDTWAPSAHARDAVYYALQFLSSVLM
NCU05242.1	MHIDIVDCQIFARAKRLLGRAIGAQDLSSATRRMKDQWAPSAKARDATFYALQFLQSVLL
FG09770.1 	MHADIVDCQMFARAKRLLGRTIGPQEFNSAQRRIKDHWAHSARARDAAFYAIKFLCSVLA
AN6733.1  	SHVDIVDCQIFAGARRLMGRAITPRDYNAAREKMVERWATKASARDATFYALKFLAECLL
          	 * ******::* *:**:**.* .:: .:: .:: : ** .* ****.:**::** . * 

MG01776.1 	PRKLTSPSMQPPVGRQTPEEPYSPQADVLMHRPWVLYIAGLIVWCYGFAIEGPCVDYIVA
NCU05242.1	PTGSGSPQSNGYS--SHMDEEYSARDDVLLNRPWVLYFAALVVWCYGYALEGACPGLPMP
FG09770.1 	PDQVSPAHAGGY----HLDEPYDARDDVLLNRPWVMYFAALVVWSYGFALEGPCPNIPLP
AN6733.1  	DHQGAH----------YEGELYCGREDYLLNRPWVIYVAALVVWCYGYALEGPIAGAPAL
          	                   * *  : * *::****:*.*.*:**.**:*:**.  .    

MG01776.1 	PTKEGRITQMQEYLHKYASVDTPEMLSEKRGMNENTALLMVLRDSFEMTRWELLHEGATL
NCU05242.1	TTHQDAVRQMREYLIKYGSIQSPEDLKSMRGINQNTALLVVLKDTFRVTRWELLHEGATL
FG09770.1 	TNKQEKLQHMRTYFIKFGTIENPADLQNRTGFNSNTALLMVLKDTFETTRWELLHEAATL
AN6733.1  	STVAEQRQDMQAFLRRVGGCREPSDLETMKGRNQCLGLLIILRDGFTNARWELLAEAANL
          	..      .*: :: : .    *  *.   * *.  .**::*:* *  :***** *.*.*

MG01776.1 	LNNCVQLNTGVLV-
NCU05242.1	LNNCILLNAGATVP
FG09770.1 	LNNCIVLNQGGTIA
AN6733.1  	LGSCIDKLREVSQ-
          	*..*:
```
